# Wet spinning of sodium carboxymethyl cellulose–sodium caseinate hydrogel fibres: relationship between rheology and spinnability†

**DOI:** 10.1039/d4sm00705k

**Published:** 2025-02-24

**Authors:** Lathika Vaniyan, Pallab Kumar Borah, Galina E. Pavlovskaya, Nick Terrill, Joshua E. S. J. Reid, Michael Boehm, Philippe Prochasson, Reed A. Nicholson, Stefan Baier, Gleb E. Yakubov

**Affiliations:** a Food Materials Research Group, University of Nottingham, Sutton Bonington LE12 5RD UK; b Sir Peter Mansfield Imaging Centre, University of Nottingham Nottingham NG7 2RD UK; c Diamond Light Source, Harwell Science and Innovation Campus Didcot OX11 0DE UK; d Motif FoodWorks Inc 27 Drydock Avenue Boston MA 02210 USA; e School of Chemical Engineering, University of Queensland Brisbane QLD 4072 Australia; f Heinz Maier-Leibnitz Zentrum, Technical University of Munich Lichtenbergstraβe 1 85748 Germany; g Food Biopolymers Laboratory, School of Food Science and Nutrition, University of Leeds Leeds LS2 9JT UK G.Yakubov@Leeds.ac.uk

## Abstract

Mimicking the fibrous structures of meat is a significant challenge as natural plant protein assemblies lack the fibrous organisation ubiquitous in mammalian muscle tissues. In this work, wet-spun hydrogel fibres resembling the anisotropic fibrous microstructure of meat are fabricated using carboxymethyl cellulose as a model polysaccharide and sodium caseinate as a model protein which are crosslinked using 1-ethyl-3-(3-dimethylaminopropyl)carbodiimide (EDC). Hydrogels and spun fibres were characterised using a combination of rheology (shear, oscillatory, and extensional), microscopy (light, polarised, and fluorescence), rheo-NMR, and X-ray diffraction. Examination of structuring behaviour under shear uncovered a relationship between enhanced biopolymer orientation along the fibre axis and a viscoelastic time-dependent ageing window for optimal hydrogel spinnability. This study provides novel rheological and structural insights into mechanisms of protein-polysaccharide assembly that may prove instrumental for development of tuneable fibres for applications in plant-based foods, tissue engineering, and biomaterials.

## Abstract 2

Mimicking the fibrous structures of meat is a significant challenge because natural plant protein assemblies lack the fibrous organisation ubiquitous in mammalian muscle tissues.

## Graphical abstract

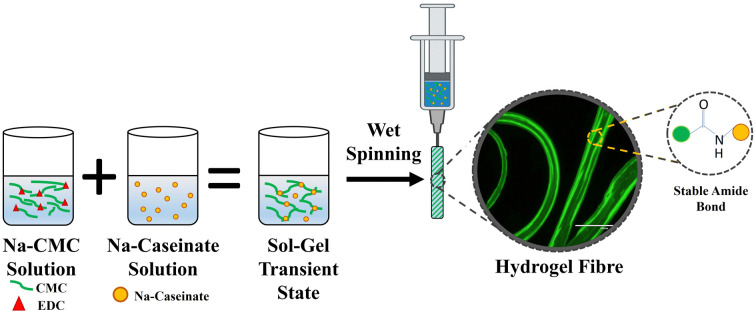

## 1. Introduction

The growing demand for sustainable, protein-rich foods requires the development of innovative technologies to mimic the texture of meat-based products using plant-based ingredients.^1,2^ The distinctive fibrous texture of meat stems from the arrangement of collagen fibres and myofibrils in the muscle tissue. However, creating analogous fibrous structures using plant-based proteins poses a marked challenge, as most commercial plant proteins, such as those derived from soy or pea, lack the inherent fibrous organisation in their native state.^3,4^ The use of hydrogel fibres is rapidly expanding across food and biomaterial applications, including the alternative meat products, where fibres have been used for imparting meat-like texture attributes to the plant-based analogue products.^5^ Equally, fibres are routinely used to design scaffolds for cellular-agriculture applications, also known as lab-grown meat.^6^ The fibre-based structures are favoured due to high versatility, tuneability, and a wide spectrum of mechanical behaviours that fibres can impart to the final products.^7,8^ In addition, the inherent ability to hold large amounts of water provides high structuring efficiency using small amounts of fibre material, thus providing cost-effective solutions for food reformulation and material design.^9^

Despite these advantages, progress in the spinning of fibres remains limited, particularly with respect to spinnability and scalability for industrial applications, hampering its widespread adoption, with the food industry being in a particularly challenging position due to stringent requirements of cost, performance, scalability, and safety. Recently, both wet and dry spinning techniques for hydrogel fibres have gained attention. For instance, Bordignon and coworkers explored the spinning of low molecular weight gels and demonstrated that altering the molecular structure of carbohydrates allows fragile hydrogels to be wet-spun, making them suitable for 3D printing applications.^10^ Lundahl and coworkers investigated the impact of shear and extensional viscosities on carbon nanofibrils and found that improved spinnability was associated with increased shear viscosity, storage modulus, and extensional viscosity.^11^ Rheological studies of polyacrylonitrile (PAN)–carbon nanotube (CNT) dispersions carried out by Lu and coworkers revealed that increasing CNT concentration enhances elastic-like behaviour and shear thinning, alongside fibre spinning performance improving for lower molecular weight PAN and similar rheological properties observed in PAN/CNT dispersions at high filler loading.^12^ Tan and coworkers^13^ studied the effects of temperature, coagulation conditions, and non-solvent on the spinnability of polyacrylonitrile-dimethyl sulfoxide solutions, showing that wet spinning is strongly influenced by the temperature and concentration of the coagulating bath, while dry spinning is primarily affected by the air gap. It was also found that addition of non-solvent such as water deteriorated the quality of wet spun fibre. Sharma and coworkers studied the extensional rheology of weakly elastic, polymeric complex fluids, by characterising their extensional relaxation time and extensional viscosity.^14–16^ By analysing the elastocapillary self-thinning, they have established the relationship between extensional relaxation time and polymer concentration.^17^

Despite these advancements, optimising fibre formation remains challenging.^18^ A particularly challenging aspect is achieving a balance between extensibility and structural integrity during the sol–gel transition.^19^ This balance should be considered across the length scales, starting from the molecular level and extending to micro- and macrostructures. Understanding the fundamental rheological and crosslinking properties that govern this transition is essential, as they ultimately determine the final structure and mechanical characteristics of fibrous hydrogels. These properties are key for final applications across foods, pharmaceuticals, and (bio)materials.

In this work, we aim to address some of these challenges. One of the key targets is to identify hydrogel formulations and crosslinking conditions where gel-setting properties are optimised to allow the formation of consistent and uniform protein-polysaccharide fibres. For this purpose, we utilise a model binary, weakly associating biopolymer system containing the sodium salt of carboxymethyl cellulose (NaCMC) and sodium caseinate (NaCas) which is crosslinked into a hydrogel using 1-ethyl-3-[3-dimethylaminopropyl]carbodiimide hydrochloride (EDC). Sodium carboxymethyl cellulose is a water-soluble polysaccharide, a cellulose derivative that is widely used in industrial applications across foods, hygiene products, pharmaceuticals and materials. This is due to tuneable and well-defined viscosifying and, more broadly, rheological properties, biocompatibility, biodegradability, and crosslinking abilities.^20–23^ NaCas is a protein derived from milk.^24,25^ Its secondary protein structure features a high content of random coil configurations (∼20%),^25,26^ which makes it effective at tethering NaCMC chains due to higher conformational flexibility^27^ (*i.e.*, as compared to tightly folded globular proteins such as, for example, lysozyme.^28^ Previous studies on carboxymethyl cellulose and sodium caseinate hydrogels have primarily focussed on the bulk gels and their viscoelastic properties.^26,29–31^ Although a wide range of cross-linking and complexation mechanisms have been explored^20,32^ which includes physical interactions,^33^ irradiation,^34^ use of multi-valent metal ions^35–37^ and low-molecular weight crosslinkers,^23,38–40^ little is known about the mechanisms of fibre formation when fibre spinning is performed under transient crosslinking conditions *i.e.*, when the cross-linking reaction is not fully completed. To modulate the conditions of the crosslinking reaction, we systematically vary the concentrations of the tethering molecule (NaCas) and the crosslinker (EDC). By decoupling the two key factors of cross-linking, *i.e.*, tether density and the speed of crosslinking, we attain the possibility of adjusting and probing the dynamic balance between extension of the polymer network in a sol state during spinning and its relaxation during the transition into a hydrogel.

We hypothesise that the emergence of anisotropic characteristics of biologic fibrous materials is associated with the alignment of polysaccharide chains in extensional flow,^41^ which is subsequently stabilised by covalent bonds between the polysaccharide and protein (*i.e.*, supressing chain relaxation upon shear cessation). To probe and scrutinise this hypothesis, we employed a range of rheological methods, including steady shear rotational rheometry, small amplitude oscillatory shear rheometry, and capillary breakup and extensional rheometry (CABER). These techniques were used to probe viscoelastic properties of the hydrogels. Polarised light microscopy has been used to reveal the effect of crosslinking conditions on the microstructure that formed during fibre spinning process. The structural characteristics on the molecular level have been probed using X-ray diffraction (XRD) and rheology coupled to sodium nuclear magnetic resonance (rheo-^23^Na-NMR) spectroscopy.^42,43^ Our results highlight the importance of rheological characteristics in the fibre spinning process and provide a deeper understanding of the factors that govern spinnability which remains underexplored, especially in the context of the controlled formation of fibrous structures with tuneable mechanical properties. As such, our work aims to provide foundational insights into designing plant-based fibrous structures for food applications, which could extend to fields like biomedical scaffolding and sustainable packaging.

## 2. Experimental

### 2.1. Materials

1-Ethyl-3-(3-dimethylaminopropyl)carbodiimide (EDC, 99%) and calcofluor white stain were purchased from Sigma-Aldrich, UK. Sodium caseinate (NaCas, ≥92% protein) was purchased from Thermo Scientific Chemicals. Sodium carboxymethyl cellulose (NaCMC) was supplied by CP Kelco, Norway (CEKOL 4000: *M*_w_, 450 kDa; DS, 0.8). Molecular weight was verified using intrinsic viscosity [*η*] measurements (details of experimental procedure is in Supplementary methods S1 and S2, ESI†). The [*η*] was found to be ∼18 dL g^−1^ in 100 mM NaCl leading to an estimated *M*_w_ of 340–470 kDa and is consistent with the information provided by the manufacturer (Supplementary Information S1 and Fig. S1, ESI†). Milli-Q water (Millipore Corp., USA) was used throughout the experiments (18.2 MΩ cm ionic purity at 25 °C). All experiments were carried out at 25 °C.

### 2.2. Preparation of NaCMC–NaCas hydrogels

Hydrogels were prepared at different concentrations of NaCas (0.1, 0.3, 0.5, 1, and 2 wt%) in 0.5 wt% NaCMC, using the ‘zero-length’ crosslinker, EDC (0, 1, 5, 10, 20 and 50 mM) (Table 1). Briefly, the mechanism of the crosslinking reaction comprises a step of: (i) EDC forming an unstable reactive ester upon interaction with a carboxyl group of the polysaccharide, and (ii) the intermediate ester then interacts with a primary amine of protein to form a peptide bond.^44^ For the formation of hydrogels, NaCas and NaCMC solutions were prepared separately in 50 mL deionized water. EDC was added to the NaCMC solution at pH 4.5 with continuous stirring for 60 s, followed by addition of the NaCas solution. The pH was lowered to 4.5 to ensure optimal EDC activity and stability of the intermediates formed during the crosslinking process.

**Table 1:** Concentrations of EDC and NaCas used with 1 wt% NaCMC

Variables	Concentrations
NaCMC	0.5%
EDC	0 mM
	1 mM
	5 mM
	10 mM
	20 mM
	50 mM
NaCas	0.1%
	0.3%
	0.5%
	1%
	2%

### 2.3. Steady-state shear and oscillatory rheology

The flow behaviour and viscoelastic properties of the hydrogel were measured on a MCR 301 rheometer (Anton Paar GmbH, Austria) equipped with a Peltier temperature control system. A concentric cylinder geometry (CC27; outer diameter, 28.9 mm; inner diameter, 26.66 mm; gap size, 1.13 mm; cone angle, 120°; effective cylinder height, 39.997 mm) was used for testing bulk solutions and gel samples. Amplitude sweeps were performed with a constant angular frequency, *ω* = 10 rad s^−1^, to identify the linear viscoelastic regime (Fig. S2, ESI†). Optimum angular frequency, *ω* and shear strain, *γ* were determined by performing frequency sweeps for sample containing NaCMC and NaCas in 1 : 1 ratio, in the frequency range, *ω* = 1–100 rad s^−1^, at constant strains of 1%. Time-dependent oscillatory shear experiments were carried out with varying concentrations of NaCas and EDC (Table 1) to identify the optimum concentrations for crosslinking and fibre spinnability. Measurements were carried out within the linear viscoelastic range to ensure that sample properties were not affected by the imposed strain or stress. Experiments were performed at a constant angular frequency, *ω* = 6.28 rad s^−1^, and constant strain, 1% for 120 min. For constructing a crosslinking diagram for biopolymer mixtures, the *G*′ values at *t* = 100 min of the reaction were plotted as a function of concentration of EDC and NaCas.

### 2.4. Capillary breakup and extensional rheology

**Fig. 1:**
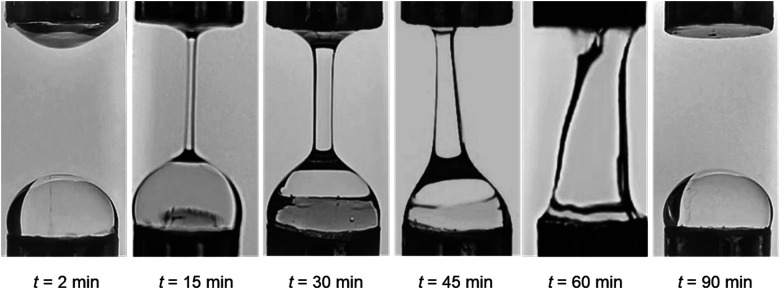
Changes in the extensional behaviour of a liquid bridge filament as a function of crosslinking time. During extensional flow measurements, the liquid sample is placed between two plates of the CaBER apparatus (plate diameter, 6 mm, initial height, *ca.* 3 mm); then the upper movable plate rapidly opens the gap, allowing the formation of a filament (final height, *ca.* 10 mm). Initially (*t* = 2 min), breakup is rapid as expected for low viscosity, Newtonian-like fluids. With the increase in crosslinking time, the filament breakup slows down, indicating the growing contribution of fluid elasticity. At *t* = 30 min of the crosslinking reaction, the filament remains sustained between the plates without breaking. As the crosslinking reaction continues, the formation of the filament represents the uniaxial extension of a viscoelastic gel. Ultimately, the strength of the gel exceeds the adhesion force between the plate and the sample, making the formation of a filament unfeasible.

Extensional capillary breakup tests were performed using a CaBER-1 extensional rheometer (Thermo Scientific Haake, Germany) equipped with an enclosed measuring unit to minimise evaporation, as described in our earlier study.^45^ For all measurements (*n* = 5), 76 μL of sample was utilised in the parallel geometry (diameter, 6 mm; initial gap, 3.01 mm, and final gap, 9.92 mm). The initial and final aspect ratio were 1 and 3.31, respectively, corresponding to a Hencky strain of 1.19. Hydrogels were tested at different time intervals to determine the effects of time-dependent ageing on crosslinking *via* analysis of capillary thinning and breakup (Fig. 1).^15,17,18^

### 2.5. Sodium rheo-NMR

A non-magnetic cone and plate geometry with 8° cone angle and 12 mm diameter (Bruker, Germany) was placed inside the 25 mm dual channel ^23^Na/^1^H resonator (Bruker, Germany) tuned to 105.68 MHz and 400.18 MHz corresponding to sodium and proton resonance frequencies, respectively. Using a specialised shaft, the assembly was positioned in the centre of the 9.4 T superconducting magnet and connected to the shear control unit (RheoSpin, Bruker, Germany). Shear rate in all rheo-NMR experiments was controlled using automated TopSpin software and maintained within ±0.01 s^−1^. The employed shear rates were 10.89, 21.00, and 30.00 s^1^. Sodium detection in rheo-NMR experiments was performed using two-dimensional triple quantum time proportional phase increment method (TQ-TPPI) with 1024 complex points sampled in the direct dimension and 512 points in the indirect dimension with 50 μs increment steps. The width of sodium 
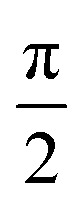
 was 50 μs, recycle delay was 100 ms, and a total time for a TQTPPI scan was under 5 min. Further details of this method are provided elsewhere.^42,46^

### 2.6. X-Ray diffraction

NaCMC–NaCas crosslinked fibres under different time-dependent ageing and crosslinking conditions were studied using an Echo D8 Advance Bruker AXS powder diffractometer (Bruker, UK) equipped with a copper tube operating at a tube voltage of 40 kV and an accelerating current of 25 mA, using Cu-Kα radiation at a wavelength of 0.1541 nm and controlled by DIFFRAC EVA software. The diffraction range (2*θ*) was 4 to 45°, with a step size of 0.02° (*n* = 3).

### 2.7. Wet spinning of hydrogel fibres

**Fig. 2:**
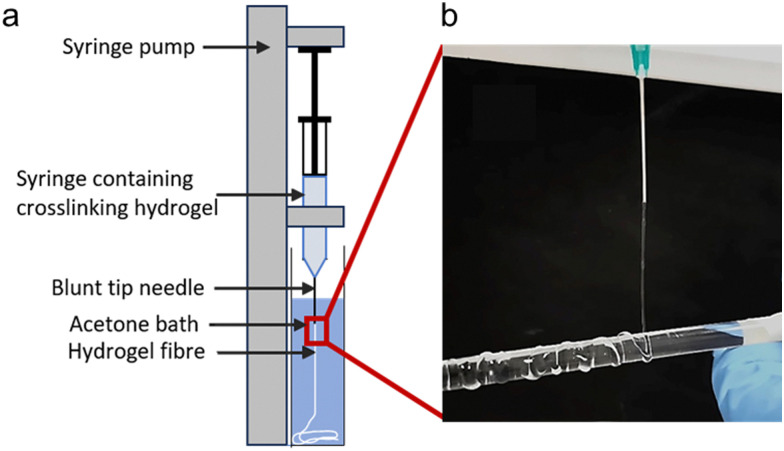
(a) Experimental set up for spinning hydrogel fibres using a syringe pump (KD Scientific, USA): (b) visual representation of hydrogel filament obtained after spinning 0.5 wt% NaCMC–NaCas solution, 30 minutes after onset of crosslinking (0.4 mL min^−1^, 21G needle, ID 800 μm).

NaCMC–NaCas fibres were spun using a syringe pump (KD Scientific, USA), as described previously by Li and coworkers,^47^ with some modifications. The experimental setup for spinning hydrogel fibres is illustrated in Fig. 2. Briefly, fibres were spun from hydrocolloid mixtures containing varying concentration of sodium caseinate (0.5, 0.75, 1 and 1.5 wt%), EDC (10, 15, 20 and 30 mM), and 0.5 wt% NaCMC. The flow rate was fixed at 0.4 mL min^−1^ for all experiments. Acetone was used as coagulant. After spinning, the fibres were air-dried. Additionally, the time-dependent ageing of hydrogels was studied using a representative hydrocolloid mixture containing 0.5 wt% of NaCMC, 0.5 wt% of NaCas (*i.e.*, 1 : 1 ratio) and 20 mM EDC).

### 2.8. Microscopy

Bright field and polarised light micrographs of the fibre samples were obtained using an Eclipse Ci-POL microscope (Nikon, Japan). Fluorescence micrographs were obtained on a EVOS FL microscope (Life technologies, USA) using the DAPI light cube (*λ*_ex_ = 350 nm; *λ*_em_ = 440). Briefly, calcofluor white stain was prepared in water at a concentration of 0.1 mg mL^−1^. Fibre samples were stained for 1 min before observation under the microscope. Mean diameter of fibres were estimated (*n* = 5) using ImageJ software (NIH, USA). Microscope images have been adjusted to improve contrast and brightness to accurately represent the data or to highlight specific features of interest. The original images are provided in the Fig. S3 and S4 (ESI†).

### 2.9. Software

SPSS interactive graphics 8.0 (1998). Chicago, III: SPSS Inc. Mateos Pérez, J. M. and Pascau, J. (2013) ‘Image processing with imageJ’. Birmingham, UK: Packt Publishing. Software library for Bruker TopSpin NMR Data files’ (2016). Washington, D.C: United States. Dept. of Energy. Origin(Pro), Version Number (Version 2022). OriginLab Corporation, Northampton, MA, USA.

## 3. Results and discussion

The ability to successfully wet-spin hydrogel fibres is highly dependent on optimising the formulation parameters, particularly the concentration of biopolymer components and the rheological properties of the precursor solution.^48^ Previous studies have demonstrated that an optimal balance of viscoelasticity, surface tension, and shear-thinning behaviour is required to ensure continuous fibre formation and structural integrity during extrusion.^12,49,50^ Building upon these insights, we explored the effect of hydrogel composition on spinnability by varying systematically the concentrations of a tether (NaCas) and a crosslinker (EDC). The choice of a model system has been instrumental in studying the relationship between viscoelastic properties of hydrogels and fibre spinnability. Using carboxymethyl cellulose (NaCMC) as a model polysaccharide and sodium caseinate (NaCas) as a model protein, we have formulated a crosslinked hydrogel system with tuneable gel-setting properties. This is achieved by independently varying the concentration of tethers that determines cross-linking density and the concentration of a crosslinker, which influences the kinetics of the crosslinking reaction (due to a size difference of approximately two orders of magnitude between EDC (∼5 Å) and NaCas (∼500 Å), it is assumed that EDC has much higher diffusivity compared to NaCas).

Our hypothesis suggests that under specific spinning conditions, the extension of polysaccharide molecules will occur, resulting in stable fibres with an anisotropic molecular and microstructure which resembles collagen and myofibril. The concept of anisotropic fibre formation is particularly important in this context, as it replicates the structural organisation observed in natural fibres like collagen, which achieve high tensile strength through their aligned molecular arrangement as reported by Li and coworkers.^7^

### 3.1. Rheological properties of the hydrogel

The rheological properties of NaCMC–NaCas crosslinked hydrogels were assessed in the concentrations ranging from semi-dilute state to concentrated solution state,^51^ covering relevant regions for technical applications. To determine the relationship between biopolymer concentration and hydrogel formation, the crosslinking process was monitored using small amplitude oscillatory shear (SAOS) rheology as a function of time. This is in agreement with the work of Lopez and coworkers.^20,51,52^ SAOS measurements yielded values of *G*′ and *G*′′ as a function of time and formulation. Monitoring the evolution of *G*′ enables assessment of the changes in crosslinking density (*v*) of the hydrogel as:^53,54^1
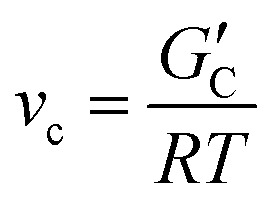
where *R* is the gas constant, *T* is the absolute temperature and 
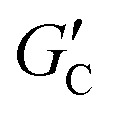
 is the storage modulus of the gel. Using the above equation, it is possible to understand the relative change in crosslinking density by normalising the storage modulus as:2
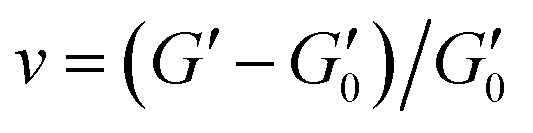
where, *G*′ is the storage modulus of the gel at time = *t* and 
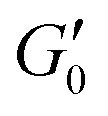
 is the storage modulus of the gel at time *t* = 0. The changes in crosslinking densities over time for various concentrations of NaCas and EDC are presented in Fig. S5 (ESI†), provide important insights into the dynamics of hydrogel formation. The storage modulus of the gel can be correlated with the strength *S*_*ω*_0__ of the gel at a given angular frequency, *ω*_0_ as:^55^3
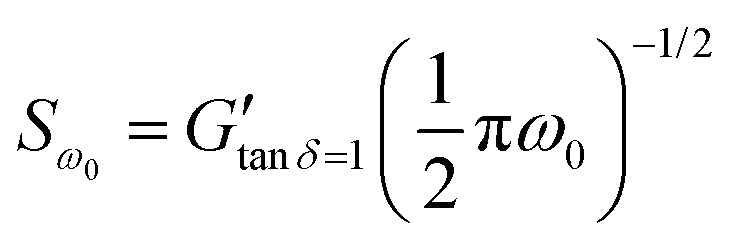
where 
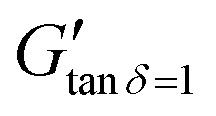
 is the value of *G*′ at the time point of its intersection with intersects *G*′′. The gel strength, *S*_*ω*_0__ is also proportional to the degree of crosslinking that enables monitoring the crosslinking reaction by measuring the corresponding time evolution of *G*′ (data not shown).

**Fig. 3:**
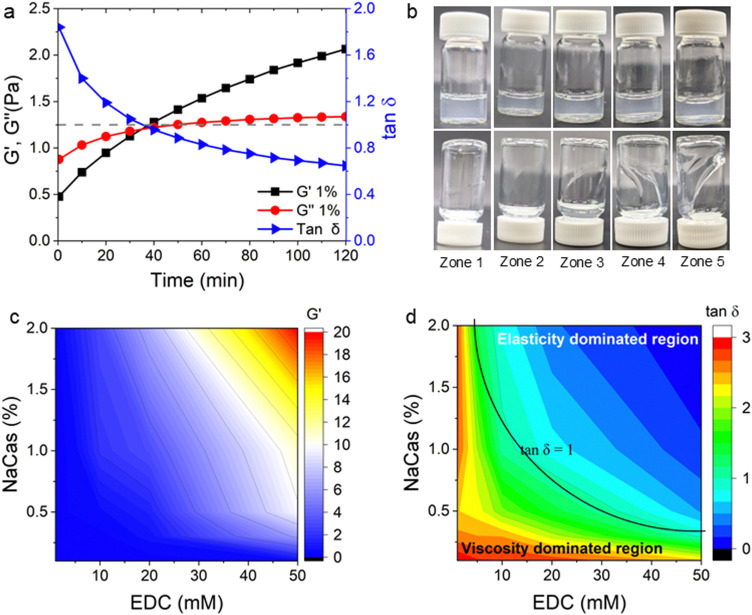
(a) Evolution of the small amplitude oscillatory shear (SAOS) rheological behaviour of NaCMC–NaCas crosslinked hydrogel (1 : 1 ratio of protein and cellulose gum) with 20 mM EDC showing changes in storage modulus (*G*′), loss modulus (*G*′′) and tan* δ* values during gelation as a function of time. The sol–gel transition time is achieved at tan* δ* = 1 when *G*′ = *G*′′. This transition time can vary depending on the concentration of crosslinking agent or concentration of biopolymers. Black dashed line is a visual guide to show tan* δ* = 1. (b) ‘protorheological’^56^ inference of (a) as a function of crosslinking time. Here, ‘zones’ correspond to the following crosslinking times: 0–20 min (zone 1), 20–40 min (zone 2), 40–60 min (zone 3), 60–80 min (zone 4), and 80–100 min (zone 5). (c) Variation in storage modulus (*G*′) with concentration of EDC and NaCas (*G*′ at *ω* = 6.28 rad s^−1^) for NaCMC–NaCas hydrogel at cross-linking time *t* = 100 min. (d) Variation in tan* δ* as a function of concentration of EDC and NaCas. Solid black line is a visual guide to denote the tan* δ* = 1 boundary.

Fig. 3a shows the corresponding values of the storage, *G*′ and loss moduli, *G*′′ as a function of reaction time for NaCMC crosslinked with NaCas mixed in 1 : 1 ratio using 20 mM EDC. Prior to the crosslinking reaction, *G*′′ is greater than *G*′, indicating that the mixture is in the free-flowing state. The crossover point of the loss and storage moduli after 30 min of reaction, represents the sol-to-gel transition and it can be used to define the onset point of crosslinking/gelation (*G*′ = *G*′′ or *G*′′/*G*′ = tan* δ* = 1). At the end of the reaction the *G*′ values were found to be significantly greater as compared to *G*′′ indicating gel-like behaviour. At this point in time, the mixture sets to form a self-supporting gel. Fig. 3a shows the tan* δ* values with respect to time and depicts that the gelation point is obtained at 35 min where tan* δ* = 1. ‘Protorheological’^56^ inference of the sample as a function of crosslinking time are shown in Fig. 3b, and clearly shows the emergence of the storage, *G*′ moduli in the gels. Here, ‘zones’ refer to the ranges of crosslinking times and compositions that represent specific phases in the hydrogel's rheo-mechanical evolution: zone 1: *G*′ ≪ *G*′′, rapid filament breakup; zone 2: *G*′ < *G*′′, slowly thinning filament; zone 3: *G*′ > *G*′′ stable filament; zone 4: *G*′ ≫ *G*′′, filament dominated by elastic extension; zone 5: *G*′ ≫ *G*′′, elastic extension.

To map the crosslinking reaction as a function of composition, the *G*′ values at *t* = 100 min of crosslinking were plotted against the concentration of NaCas and EDC. The variation in the *G*′ values over time with increasing EDC concentrations is plotted in the Fig. S6 (ESI†). Fig. 3c shows the comparison of storage modulus at *t* = 100 min with increasing concentrations of both NaCas and EDC. The variation in *G*′ and *G*′′ over time for different of NaCas concentrations, specific to each EDC concentration are plotted separately in the Fig. S7 (ESI†). In un-crosslinked systems the loss modulus (*G*′′) value was greater than the storage modulus (*G*′). As the gel crosslinks the storage modulus increases (*G*′′ < *G*′). It was observed that crosslinking occurred at a minimum concentration of 10 mM EDC with 1 wt% NaCas and 50 mM EDC with 0.3 wt% NaCas. The storage modulus and crosslinking density increase in tandem with increasing concentration of EDC and NaCas. Fig. 3d shows that the tan* δ* value decreased with increasing concentration of EDC and NaCas indicating the formation of crosslinked gel. At low concentrations of NaCas and EDC, there was a delay in the onset of crosslinking, while at higher concentrations, the gel sets rapidly, leaving very little window for the fibres to be spun. The optimum concentration for favourable gel setting behaviour was obtained with a 1 : 1 ratio of NaCMC to NaCas and 20 mM EDC.

**Fig. 4:**
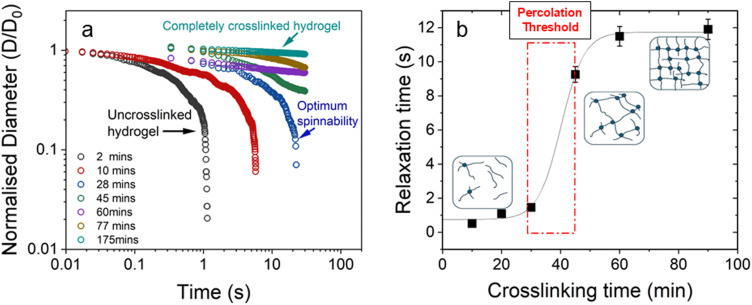
(a) Normalised filament diameter as a function of time for crosslinking hydrogel with a total polymer concentration of 1 wt% and 20 mM EDC. Early filament thinning and breakage was observed in weakly crosslinked polymer while completely crosslinked polymers exhibited no filament formation. (b) Characteristic relaxation time (*λ*_E_) from CaBER experiments as a function of crosslinking time obtained by fitting the exponential phase of CaBER data. Red dashed box is a visual guide to indicate the evidence of percolation threshold behaviour. Error bars represent *n* = 5. Fitted curves for extensional relaxation time, *λ*_E_ are shown in Fig. S8 (ESI†).

We also studied the extensional flow behaviour of the hydrogels using capillary breakup and extensional rheology (CaBER).^57^ Capillary breakup is widely understood as a surface tension induced breakup of filaments at low concentrations of the crosslinked solution which determines the lower limit of spinnability.^58,59^ The time *t* = 0 is defined as the time at which the upper plate has reached its final position (Hencky strain, 1.19). It was observed that the hydrogel starts crosslinking immediately after sample preparation and forms a highly viscous gel within two hours of preparation. The gel showed marked extensional properties for a long period of time at a concentration of NaCMC and NaCas at 1 : 1 ratio with 20 mM of EDC. The gel formed is highly flexible within the range of 30 min to 60 min after which it sets completely. Fig. 4a shows the evolution of fibre diameter on the CaBER with respect to time. The extensional relaxation time, *λ*_E_ were determined from the exponential function (at the initial stages of capillary thinning) as:^60^4
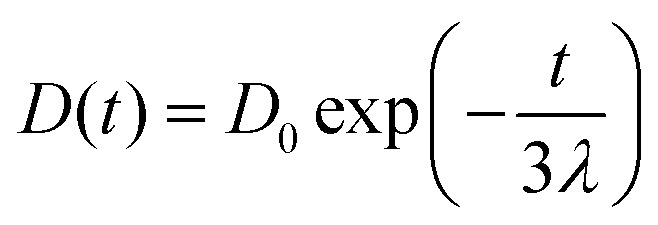
where, *D* is the diameter of the thinning capillary and *D*_0_ is the diameter of the thinning capillary at time, *t* = 0. One can clearly observe the evolution of the hydrogel from completely un-crosslinked to completely crosslinked as a function of time. Changes in relaxation time of the gel in response to crosslinking time provides further evidence of percolation and three-dimensional network formation as the sol-to-gel transformation takes place,^61^ and is shown in Fig. 4b. When *G*′′ > *G*′ in the system, the gel network is weakly formed, and the relaxation time is lower than the percolation threshold value (*r* < *r*_c_). When *r* > *r*_c_; *G*′ > *G*′′ and a plateau region is observed, providing indications that the polymer network has completely developed. We have provided a visual depiction of the network formation as a function of the crosslinking time within insets in Fig. 4b.

### 3.2. Microstructural analysis of hydrogel fibres

**Fig. 5:**
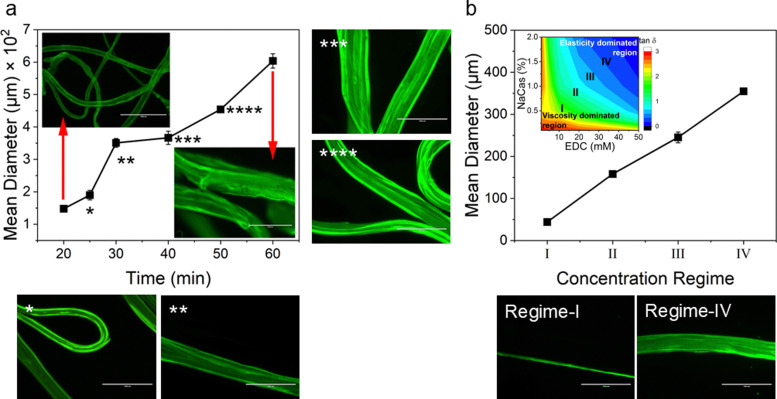
(a) Images of hydrogel fibres formed at different crosslinking times viewed using an EVOS fluorescent microscope. Fibre diameters were calculated using ImageJ (NIH, USA). *Star marks within the plot are relative to the corresponding *marks in the associated fluorescent micrographs. (b) Fibre diameter in different concentration regimes. The weak hydrogels formed at lower concentrations resulted in thin filaments. By contrast, rapid gelation at higher polymer concentrations led to the formation of fibres with irregular thickness. Inset is a rendition of Fig. 3d, but with added demarcations for different regimes. Regimes I–IV indicates transition from viscosity-dominated to elasticity-dominated as a function of NaCas and EDC concentrations. Fluorescent micrographs show spun fibres at regimes I and IV.

Hydrogel fibres were spun at a constant flow rate of 0.4 mL min^−1^ at different crosslinking times using a syringe pump. Microscopic images of EDC crosslinked NaCMC–NaCas fibres were analysed to determine the average diameter of the fibres spun at different time intervals. Fig. 5a displays the variation in diameters of fibres spun at various time intervals following the initiation of crosslinking. The results show that the fibre diameter increased with the increase in polymer concentration and crosslinking time. The variation in fibre diameter at different concentration regimes is displayed in Fig. 5b. Smooth fibres were obtained by wet spinning NaCMC–NaCas hydrogel within the interval of optimum spinnability as discussed in Section 3.1. These changes can be attributed to the increase in the extensional viscosity of the biopolymer mixture. The wet spun hydrogel fibres were coagulated in an acetone bath. This resulted in dehydration of the gel-fibres. After air drying, the resulting fibres had a white, lustrous appearance.

**Fig. 6:**
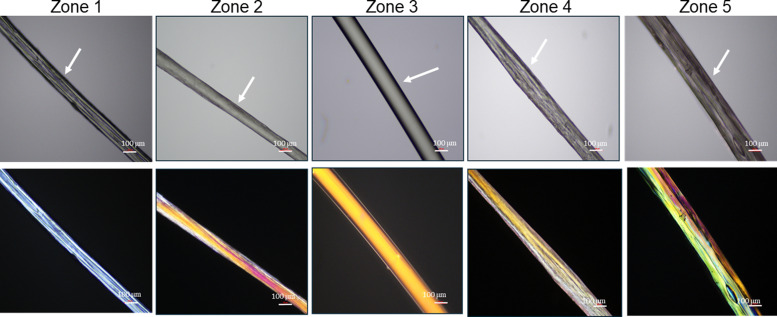
Polarised optical micrographs 
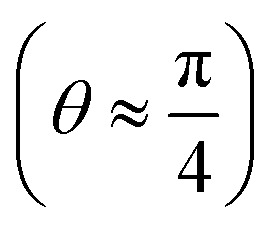
 of crosslinked NaCMC–NaCas fibres spun at different rheo-mechanical zones during crosslinking. Zones correspond to crosslinking time as: 0–20 min (zone 1), 20–40 min (zone 2), 40–60 min (zone 3), 60–80 min (zone 4), and 80–100 min (zone 5). White arrows are a visual guide indicating Becke lines. Scale bar is 100 μm. 
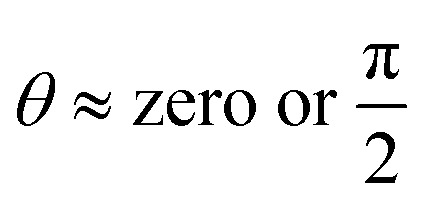
 are shown in Fig. S9 (ESI†).

The polarized images of NaCMC–NaCas crosslinked fibres alongside comparative optical micrographs at different rheo-mechanical zones during the crosslinking process are shown in Fig. 6. Refractive index of the spun fibres was greater than air, evidenced by the presence of Becke lines with positive relief (Fig. 6, highlighted by arrows). Increased crosslinking time results in thicker fibres and the surface of the fibre transitioned from smooth to irregular with scalloped edges. As can be seen, within the zones of optimum spinnability (particularly zone 3), fibres demonstrated strong birefringence, *i.e.*, anisotropy in the transmission of light. The fibre appears to have a principle linear optical axis along the major axis, where the polarised light is disintegrated into slow and fast components which were not in phase with each other. Note, the extinction events upon rotation of the sample stage are shown in Fig. S9 (ESI†). Here, the transmission intensity is a function of the angle, *θ* that the fibre principal linear axis makes with the axis of polarisation; extinction is observed when 
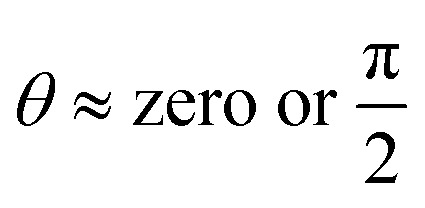
, whereas transmission is high when 
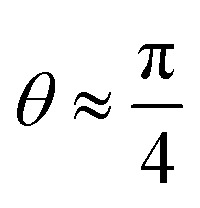
 (transmitted light intensity ≈ sin^2^(2*θ*)). Fibres within zones 1, 2, and 5 did not demonstrate extinction as a function of *θ*, whereas a strong effect of *θ* was observed for fibres within zones 3 and 4; the latter is indicative of fibre anisotropy plausibly with nematic ordering. Previous studies have shown similar results for fibril alignment in collagen fibres.^62,63^

### 3.3. Short-range molecular order of hydrogel fibres

**Fig. 7:**
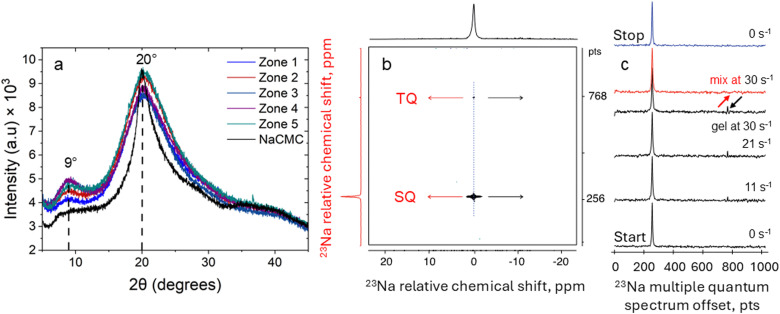
(a) X-Ray diffraction curves for NaCMC and hydrogel fibres spun at different crosslinking times and rheo-mechanical zones: 0–20 min (zone 1), 20–40 min (zone 2), 40–60 min (zone 3), 60–80 min (zone 4) and 80–100 min (zone 5). Dashed lines are a visual guide to denote the diffraction peaks. NaCMC, sodium carboxymethyl cellulose. (b) ^23^Na TQ-TPPI spectra of 1 wt% NaCMC–NaCas crosslinked gels: 2D TQ-TPPI map measured under 30 s^−1^ with all relative terminology. Dotted blue line is a visual guide denoting the sodium chemical shift frequency. (c) ^23^Na multiple quantum (MQ) spectra taken from 2D maps as shown by dashed blue line in (b) at 0 s^−1^, 11 s^−1^, 20 s^−1^, 30 s^−1^, and post shear. MQ spectrum of mix solution of biopolymers without crosslinking agent added and sheared at 30 s^−1^ is shown in red. Please note the absence of TQ peak at the highest shear rate as shown by arrows.

Powder X-ray diffraction (XRD) enables ascertaining molecular orientation in biopolymer fibres and was utilised to characterise such systems as spider silk^64^ and binary polysaccharide gels based on xanthan gum.^65^ In this work XRD is used to investigate the emergence of molecular ordering in the NaCMC–NaCas crosslinked fibres as a function of crosslinking time. Fig. 7a shows XRD diffraction patterns of pure NaCMC and hydrogel fibres spun at different zones of the sol–gel transition. The data show the presence of a smaller peak at 2*θ* ≈ 9° and a larger peak at 2*θ* ≈ 20°. The peak at 2*θ* ≈ 20° can be primarily attributed to the intermolecular spacing in the amorphous NaCMC.^37,53,66^

For the hydrogel fibres, this peak becomes broader, indicating wider distribution of intermolecular distances. Notably, a marked increase in peak intensity at 2*θ* ≈ 9° was observed for the hydrogel fibres compared to NaCMC alone, as well as compared to fibres spun in zone 1. Our findings align with previous research conducted by Souza and coworkers.^53^ The latter are equivalent to quiescently cross-linked gels, because NaCMC chains in these fibres have sufficient time to relax before cross-linking takes place. Thus, we suggest that the peak at 2*θ* ≈ 9° is associated with longer range structure, which gets accentuated by the spinning process during fibre manufacture. The ratio of peak areas was used to estimate the degree of long-range structuring as shown in Table 2. The fibres spun within zone 3 showed the highest degree of long-range structuring, followed closely by zone 4 with the peak intensity ratio of ∼0.1 compared to 0 for pure NaCMC. Although specific details of long-range structuring require further analysis, it is evident that XRD findings appear to be consistent with the results obtained from polarised microscopy.

**Table 2:** Peak areas and the ratio of area of peaks at 2*θ* ≈ 9° to 2*θ* ≈ 20°

Zone	Peak 1	Peak 2	Ratio
NaCMC	0	14 857.73	0
1	270.48	31 116.51	0.0087
2	1279.19	38 632.77	0.0331
3	2404.99	23 910.67	0.1006
4	2911.11	29 348.14	0.0992
5	1856.37	32 880.64	0.0565

To probe the hydrogel structure further, we used sodium rheo-NMR – a versatile and sensitive technique which provides insights into molecular alignment within sodium-containing hydrogel networks under shear.^46^ Since both our biopolymers, NaCas and NaCMC, contain Na ions, ^23^Na rheo-NMR would provide a means of distinguishing between free and bound sodium ions.^42^ The triple quantum (TQ) coherence, contribution to sodium NMR signal arises from slow-motion states of sodium ions as compared to the freely diffusing ones. Such slow-motion states can arise, for example, from electrostatic binding of sodium ions to hydrogel macromolecules. This interaction can be detected using a two-dimensional (2D) scan induced through specific ^23^Na TQ-TPPI MR protocol, employed in this work. Fig. 7b shows the 2D ^23^Na TQ-TPPI spectrum, where sodium chemical shift is shown in the direct (horizontal) dimension, while sodium multiple quantum spectra are shown in the indirect (vertical) dimension that separates the single quantum and triple quantum coherences. Analysis of sodium multiple quantum spectra taken at the sodium chemical shift frequency (dotted blue line in Fig. 7b) enables monitoring the degree of association of sodium cations with hydrogel biomolecules by analysing the intensity of TQ population under shear. Fig. 7c shows sodium multiple quantum (MQ) spectra produced in this manner in the gel under pre-shear, sheared at 11 s^−1^, 20 s^−1^, 30 s^−1^ and post shear conditions. No TQ sodium peaks were detected in the absence of shear. This indicates an isotropic environment of the static crosslinked gel as probed by sodium cations. The introduction of shear, however, results in the emergence of distinct TQ peaks in the sheared gel system as seen in Fig. 7c (highlighted with black arrow).

We postulate the emergence of the ^23^Na TQ signals to the molecular order or molecular alignment formed in the gel at the onset of shear. Tests were also performed on a mixed solution of NaCMC and NaCas, without the addition of the crosslinking agent. As can be seen from Fig. 7c (MQ spectrum shown in red), no TQ sodium signal was detected in the mixed biopolymer system under shear. This indicates that molecular alignment must be emerging at the interface of shear gel particles, most likely in a form of an electrical double layer. In the mixed, un-crosslinked biopolymer system the ionic interactions lack a distinct interface, making their relaxation more random than in the case of an interfacial layer of aligned counter-ions. During shear, the requirement of electroneutrality generates a streaming potential in the direction of flow that separates sodium ions between those in a slow-motion state and freely diffusing ones. Similar effects have been observed before in other biopolymeric fluids^46^ where the formation of molecular order was confirmed by detection of sodium residual quadrupolar coupling constant. Although the likely location of the sodium ions in slow-motion states is at the interface between shear-gel particles, it is possible to propose an alternative localisation of electrokinetically trapped states. Considering small amplitudes of TQ signals, the effects of gel friction against the walls of the measuring geometry cannot be excluded.^46^

In summary, the results of this study reveal a clear relationship between the rheological properties of covalently crosslinked NaCMC–NaCas hydrogels and their spinnability. The observed trends suggest that specific rheological parameters – such as viscoelasticity, precursor concentrations, sol–gel transition time, and polymer relaxation behaviour – play critical roles in determining molecular alignment and orientation during the fibre spinning process. In this context, a suite of quantifiable parameters provides a valuable toolbox for optimising spinning conditions and enabling the rational design of biopolymer fibres.

## 4. Conclusions

In this study, a model protein-polysaccharide hydrogel system was designed to probe the effects of crosslinking on fibre spinnability and structural alignment during extension. The EDC-mediated crosslinking of NaCMC and NaCas resulted in the formation of homogenous gel. Hydrogels were prepared in different ratios by varying the concentrations of EDC and NaCas at a constant concentration of NaCMC, 1.0 wt%. By exploring capillary breakup and rheological behaviour using small amplitude oscillatory shear rheology, a direct connection between the rheological properties of hydrogels and fibre spinnability was uncovered. This study demonstrates that fibre spinnability is intricately linked to the evolution of viscoelastic properties and is influenced by the sol–gel transition time. During the sol-to-gel transition, the dynamic properties of the transient molecular networks facilitate the spinning of highly regular and homogeneous fibres. Specifically, for the 1 wt% NaCMC–NaCas hydrogel, optimal spinnability was observed between 40 and 60 min into the crosslinking reaction, where tan *δ* ≈ 1. Notably, evidence of shear-induced anisotropic alignment of polysaccharide chains was identified. This alignment was characterized using polarized light microscopy, XRD, and rheo-NMR.

Overall, our findings provide a foundational platform for future studies. By exploring the fundamental principles governing spinnability and through careful tailoring of the crosslinking conditions, we have uncovered a dynamic balance between extension and relaxation of weakly associated polymer networks to form aligned fibres. These insights will help guiding the development and optimisation of materials tailored to specific applications in fibre spinnability across diverse fields, including applications in foods, drug delivery, wound care scaffolding, tissue engineering,^67^ and sustainable packaging. From our perspective, the insights generated using a model hydrocolloid system will enable the development of edible fibres designed with food-grade hydrocolloids and food-compatible crosslinking agents.

## Abbreviations

DS: Degree of substitution
EDC: 1-Ethyl-3-(3-dimethylaminopropyl)carbodiimide
*G*′: Storage modulus
*G*′′: Loss modulus
NaCMC: Sodium carboxymethyl cellulose
NaCas: Sodium caseinate
Rheo-NMR: Rheology-nuclear magnetic resonance
tan *δ*: Loss factor
TQF: Triple quantum filter
TPPI: Time-proportional phase incrementation
XRD: X-Ray diffraction
*λ*_E_
: Extensional relaxation time
*ω*
: Angular frequency

## Author contributions

Lathika Vaniyan, conceptualisation, data curation, formal analysis, investigation, validation, visualisation, writing – original draft, writing – reviewing & editing; Pallab Kumar Borah, formal analysis, investigation, methodology, visualisation, writing – reviewing & editing; Galina E. Pavlovskaya, data curation, formal analysis, investigation, validation, resources, writing – reviewing & editing; Nick Terrill, formal analysis, investigation, validation, writing – reviewing & editing; Joshua E. S. J. Reid, investigation, writing – reviewing & editing; Michael Boehm, conceptualisation, investigation, writing – reviewing & editing; Philippe Prochasson, investigation, writing – reviewing & editing; Reed A. Nicholson, investigation, formal analysis, writing – reviewing & editing; Stefan Baier, conceptualisation, investigation, formal analysis, funding acquisition, writing – reviewing & editing; Gleb E. Yakubov, conceptualisation, methodology, formal analysis, investigation, funding acquisition, resources, supervision, project administration, writing – reviewing & editing. All authors reviewed the manuscript.

## Data availability

The data supporting this article has been included as part of the ESI.†

## Conflicts of interest

The authors declare that they have no competing financial interests or personal relationships that could have appeared to influence the work reported in this paper.

## Supplementary Material

SM-021-D4SM00705K-s001
