# Correction: Wet spinning of sodium carboxymethyl cellulose–sodium caseinate hydrogel fibres: relationship between rheology and spinnability

**DOI:** 10.1039/d5sm90047f

**Published:** 2025-03-26

**Authors:** Lathika Vaniyan, Pallab Kumar Borah, Galina E. Pavlovskaya, Nick Terrill, Joshua E. S. J. Reid, Michael Boehm, Philippe Prochasson, Reed A. Nicholson, Stefan Baier, Gleb E. Yakubov

**Affiliations:** a Food Materials Research Group, University of Nottingham Sutton Bonington LE12 5RD UK; b Sir Peter Mansfield Imaging Centre, University of Nottingham Nottingham NG7 2RD UK; c Diamond Light Source, Harwell Science and Innovation Campus Didcot OX11 0DE UK; d Motif FoodWorks Inc. 27 Drydock Avenue Boston MA 02210 USA; e School of Chemical Engineering, University of Queensland Brisbane QLD 4072 Australia; f Heinz Maier-Leibnitz Zentrum, Technical University of Munich Lichtenbergstraβe 1 85748 Germany; g Food Biopolymers Laboratory, School of Food Science and Nutrition, University of Leeds Leeds LS2 9JT UK G.Yakubov@Leeds.ac.uk

## Abstract

Correction for ‘Wet spinning of sodium carboxymethyl cellulose–sodium caseinate hydrogel fibres: relationship between rheology and spinnability’ by Lathika Vaniyan *et al.*, *Soft Matter*, 2025, https://doi.org/10.1039/d4sm00705k.

The authors regret an error in Fig. 4 in the original manuscript. The correct version of Fig. 4 is as shown below.

**Fig. 1:**
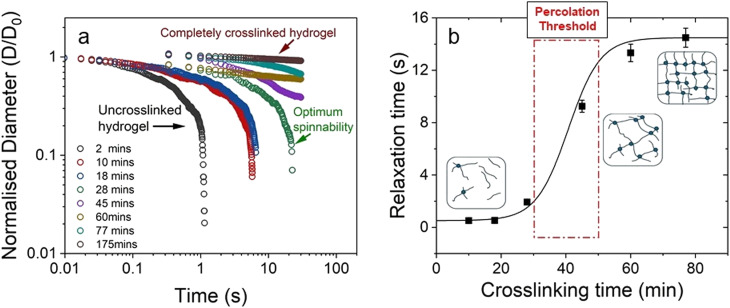
(a) Normalised filament diameter as a function of time for crosslinking hydrogel with a total polymer concentration of 1 wt% and 20 mM EDC. Early filament thinning and breakage was observed in weakly crosslinked polymer while completely crosslinked polymers exhibited no filament formation. (b) Characteristic relaxation time (*λ*_E_) from CaBER experiments as a function of crosslinking time obtained by fitting the exponential phase of CaBER data. Red dashed box is a visual guide to indicate the evidence of percolation threshold behaviour. Error bars represent *n* = 5. Fitted curves for extensional relaxation time, *λ*_E_, are shown in Fig. S8.

The Royal Society of Chemistry apologises for these errors and any consequent inconvenience to authors and readers.

