# Supplementary material for: Wet spinning of sodium carboxymethyl cellulose–sodium caseinate hydrogel fibres: relationship between rheology and spinnability
Source: Soft Matter. 2025 Feb 24;21(20):3946–56. doi: 10.1039/d4sm00705k (PMC11852227; doi:10.1039/d4sm00705k)
Supplement: SM-021-D4SM00705K-s001 [file SM-021-D4SM00705K-s001.pdf]

Supplementary Information (SI) for Soft Matter.
This journal is © The Royal Society of Chemistry 2025

Supporting Information

Wet Spinning of Sodium Carboxymethyl Cellulose - Sodium Caseinate Hydrogel Fibres: Relationship between Rheology and Spinnability

Lathika Vaniyan[a], Pallab Kumar Borah[a,f], Galina E. Pavlovskaya[b], Nick Terrill[c], Joshua E.S.J. Reid[a], Michael Boehm[d], Philippe Prochasson[d], Reed A. Nicholson[d], Stefan Baier[d,e], Gleb E. Yakubov*[a,g]

[a]*Food Materials Research Group, University of Nottingham, Sutton Bonington, LE12 5RD, United Kingdom*

[b]*Sir Peter Mansfield Imaging Centre, University of Nottingham, Nottingham, NG7 2RD, United Kingdom*

[c]*Diamond Light Source, Harwell Science and Innovation Campus, Didcot, OX11 0DE, United Kingdom*

[d]*Motif FoodWorks Inc, 27 Drydock Avenue, Boston, MA 02210, USA.*

[e]*School of Chemical Engineering, University of Queensland, Brisbane, QLD 4072, Australia.*

[f]*Heinz Maier-Leibnitz Zentrum, Technical University of Munich, Lichtenbergstraβe 1, 85748, Germany*

[g] *Food Biopolymers Laboratory, School of Food Science and Nutrition, University of Leeds, Leeds, LS2 9JT, United Kingdom*

*Corresponding author: Gleb E. Yakubov, Professor of Food Biopolymers, School of Food Science and Nutrition, University of Leeds, Leeds, LS2 9JT, United Kingdom.

S1. Viscosity Measurements

NaCMC samples with varying concentrations (0.01%, 0.05%, and 0.1% w/v) were prepared in Milli-Q water, as well as in different concentrations of sodium chloride (10 mM, 30 mM, and 100 mM) to adjust the ionic strength[1, 2] and phosphate buffer (10 mM, 30 mM, and 100 mM) in order to keep the pH constant. The solutions were stirred overnight to ensure complete hydration of the NaCMC[3]. The viscosity of the solutions was measured using a MCR 301 rheometer (Anton Paar GmbH, Austria) equipped with a Peltier temperature control system. A concentric cylinder geometry was utilised (CC27; outer diameter: 28.9 mm; inner diameter: 26.66 mm; gap size: 1.13 mm; cone angle: 120°; effective cylinder height: 39.997 mm). From the kinematic viscosities, the relative, specific, and reduced viscosities of the NaCMC solutions were calculated at each concentration at 25 °C [3]. Finally, the intrinsic viscosities $[\eta]$ were determined with the use of Solomon-Ciuta equation (please see section S3). The intrinsic viscosity values for 100 mM NaCl solvent show highest suppression of non-ideality, as evident from the weakest dependence of $[\eta]$ on concentration ($[\eta]$ = 20.1±0.8 dL/g). This value was used to estimate the molecular weight (M_W) of NaCMC using Bohdanecky relation[4, 5] and the values of the persistence length (L_p=14.3 nm) reported in literature[3]. as:

$$\left(\frac{M_w^2}{[\eta]}\right)^{1/3} = A_0 M_L \phi^{-1/3} + B_0 \phi^{-1/3} \left(\frac{2L_P}{M_L}\right)^{-1/2} M_W^{1/2}$$

where Φ is the Flory–Fox constant (2.86×10^{23} mol^{-1}) and A_0 = 1.053 and B_0 = 1.016 are tabulated coefficients[5]. The estimated values of M_W were found to be 340 ± 20 kDa. For the lower limits of the L_p = 12 nm, the estimated values of M_W were found to be ~470 kDa. The determined range of molecular weights appears to be consistent with manufacturer specifications.

S2. Intrinsic viscosity

NaCMC has been extensively studied for its behaviour in various environments including solubility, viscosity, and interaction with other substances[6-8]. The intrinsic viscosity $[\eta]$ was calculated using Solomon-Ciuta equation[9] as:

$$[\eta] = \frac{1}{c}\sqrt{2\eta_{sp} \square 2ln\eta_{rel}}$$

where η_{sp} is the specific viscosity, η_{rel} is the relative viscosity, and c is the polymer concentration (in g/dL).

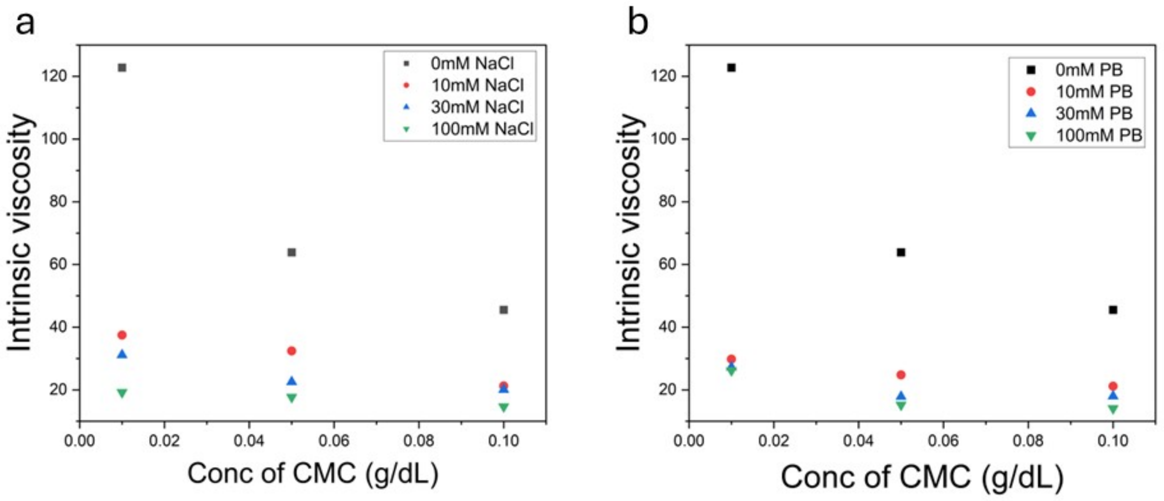

Figure S1: Intrinsic viscosity data for varying concentrations of CMC in different concentrations of NaCl and phosphate buffer.

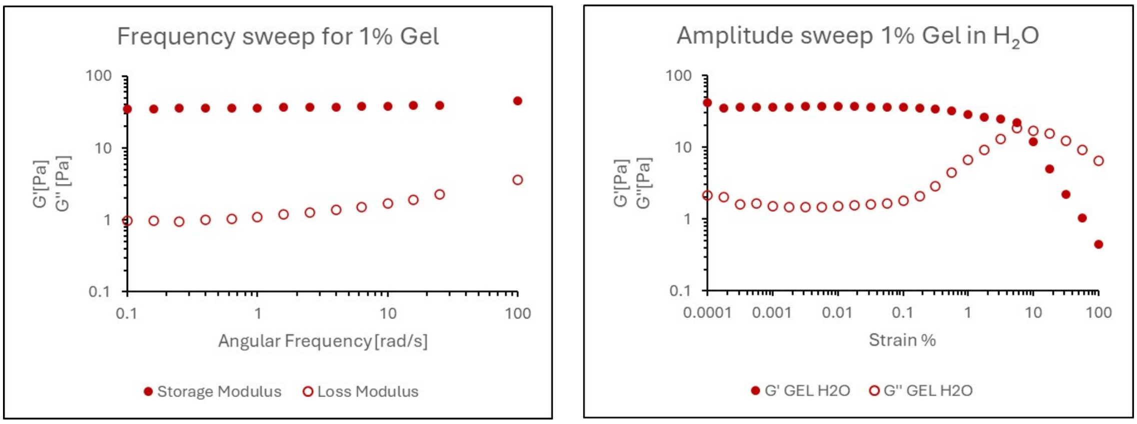

Figure S2: Oscillatory rheometry of 0.5%/0.5% NaCMC/NaCas: frequency (left panel) and amplitude sweeps (right panel).

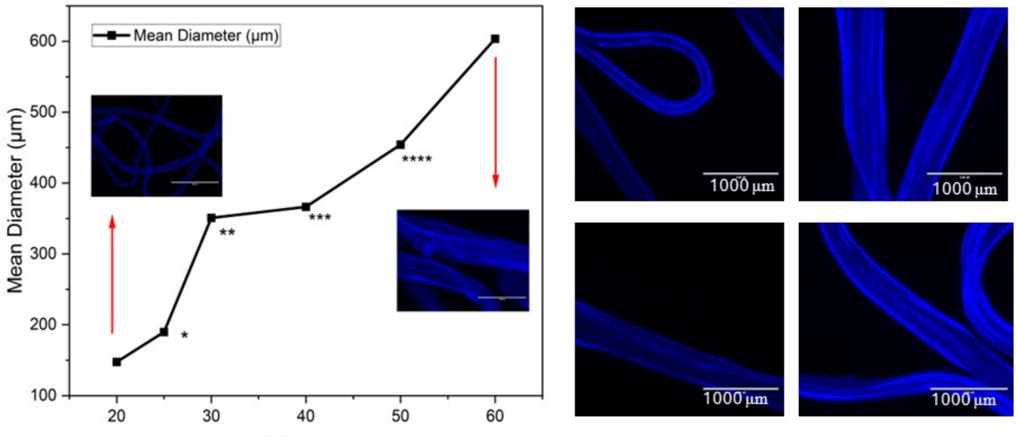

Figure S3: Images of hydrogel fibres formed at different cross-linking times, viewed using an EVOS fluorescent microscope. Fibre diameters were calculated using ImageJ (NIH, USA).

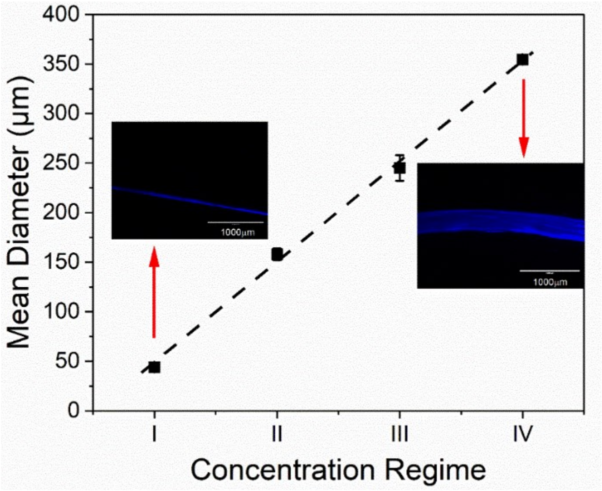

Figure S4. Fibre diameter in different concentration regimes. The weak hydrogels formed at lower concentrations resulted in thin filaments. By contrast, rapid gelation at higher polymer concentrations led to the formation of fibres with irregular thickness.

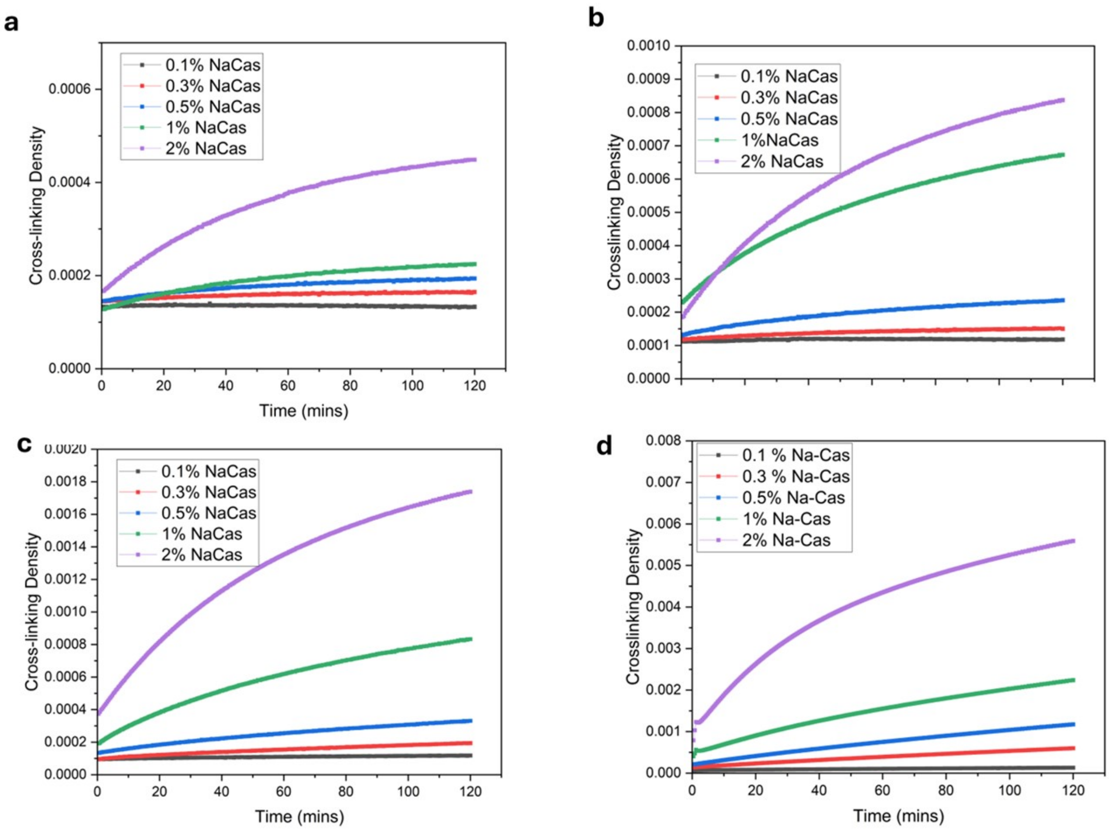

Figure S5: Crosslinking density as a function of time in the presence of EDC at various concentrations: (a) 5 mM; (b) 10 mM; (c) 20 mM; (d) 50 mM.

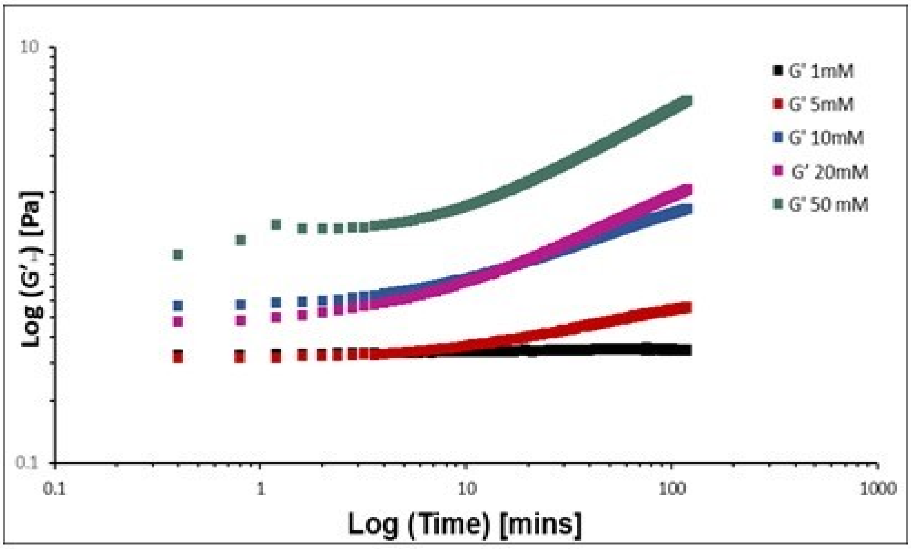

Figure S6: Change in *G'* values of NaCMC-NaCas (1:1 ratio) with respect to time at increasing EDC concentrations.

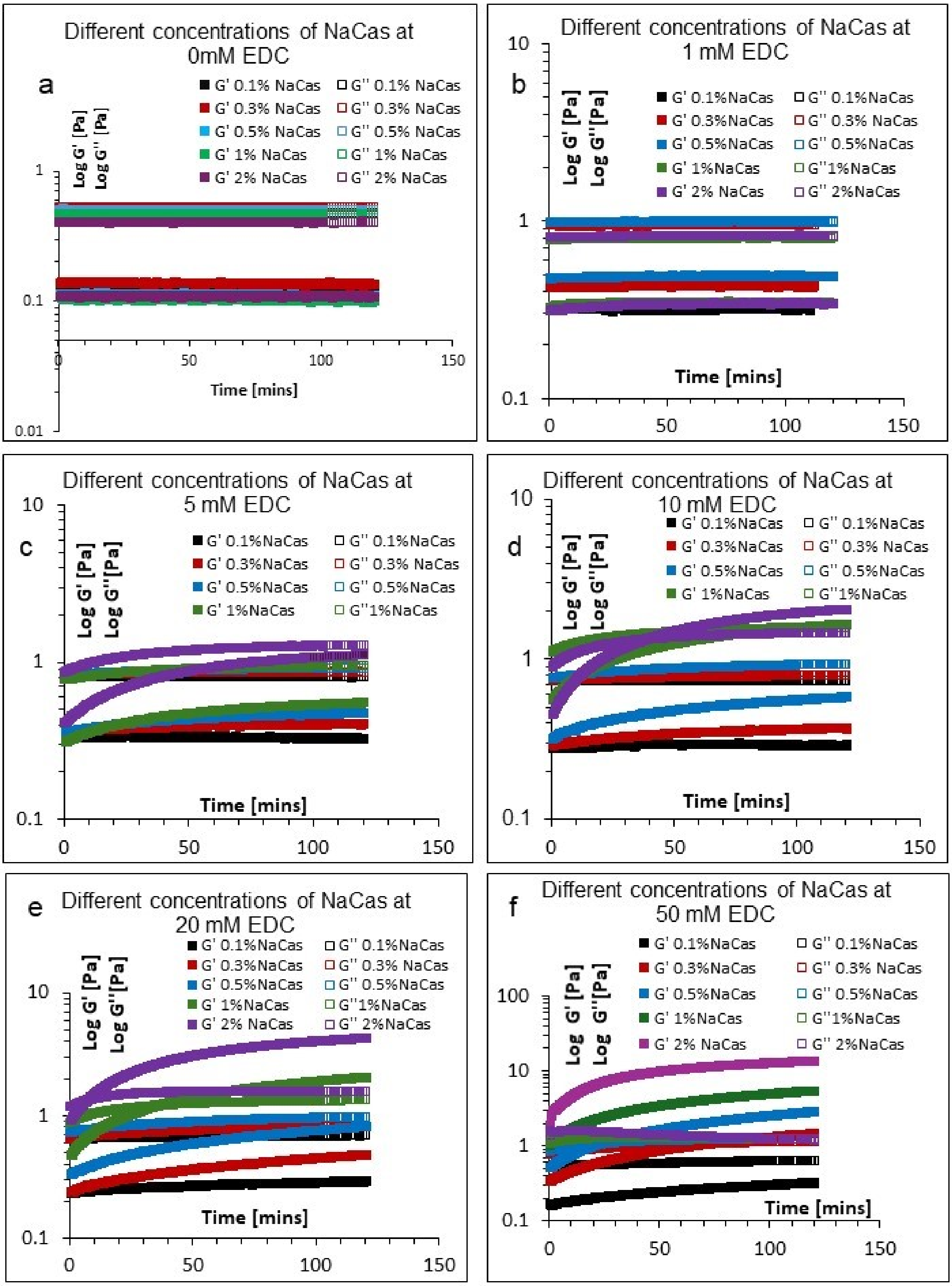

Figure S7: *G'* and *G''* values for different concentrations of NaCas with respect to time, in the presence of EDC at various concentrations: (a) 0 mM, (b) 1 mM, (c) 5 mM, (d) 10 mM, (e) 20 mM, (f) 50 mM.

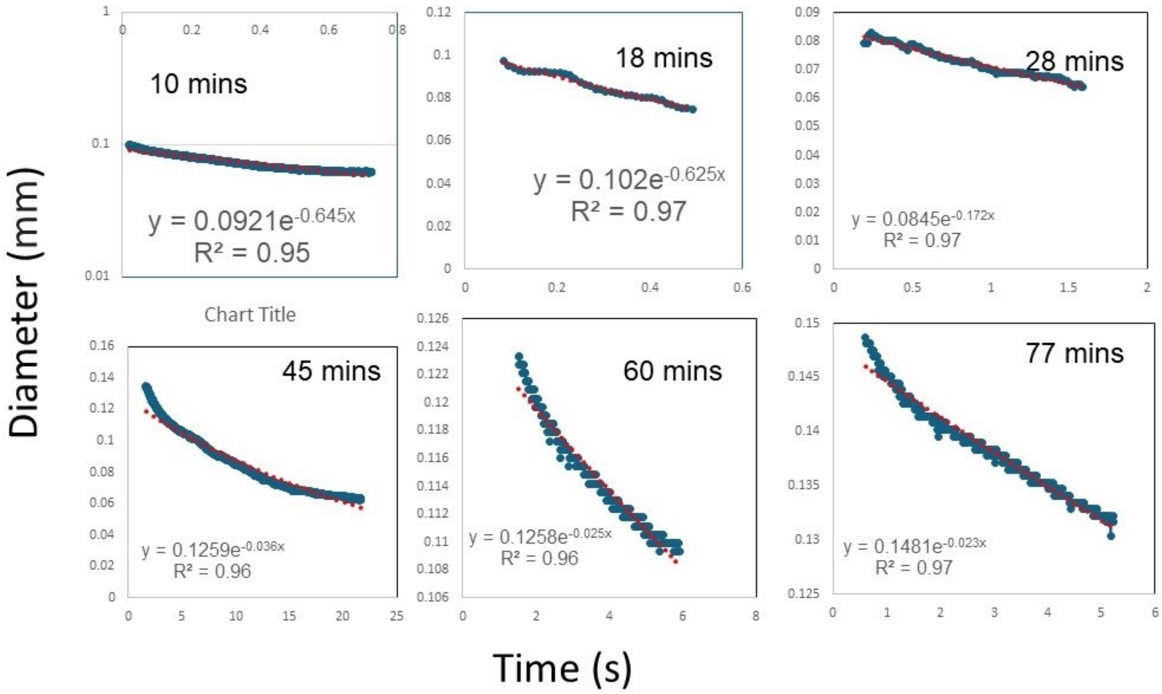

91

92 **Figure S8:** Fitted Curves for relaxation time. Red dotted line shows exponential fitting.

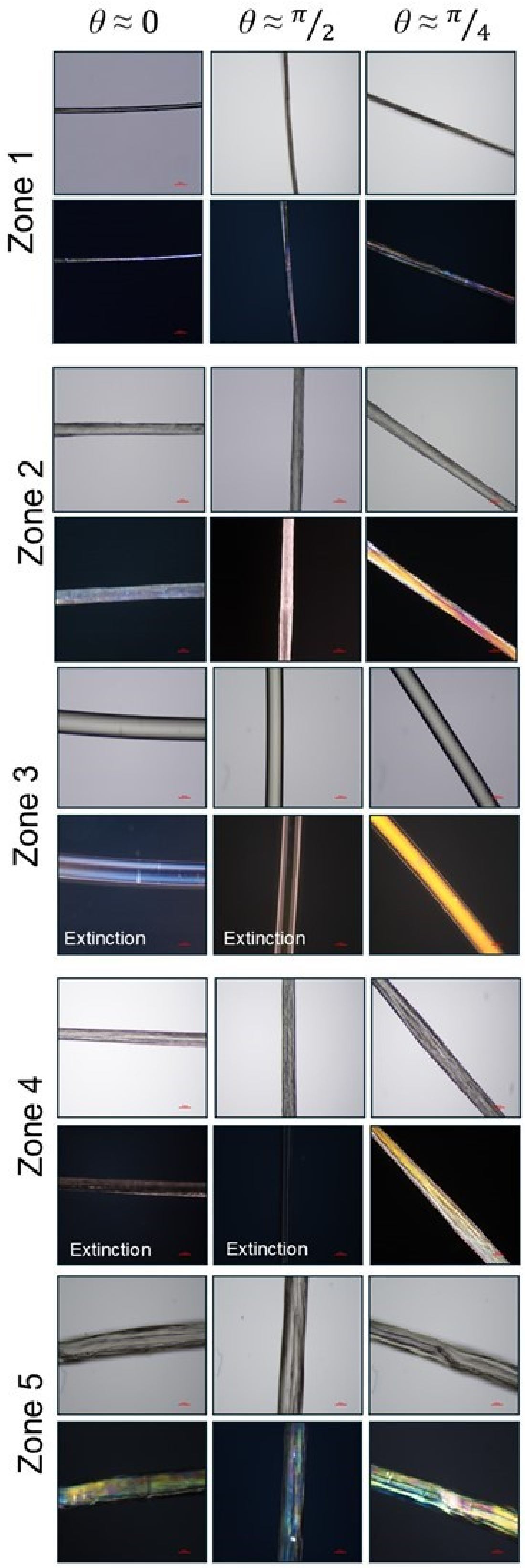

Figure S9. Fibre diameter in different zones. The weak hydrogels formed at lower concentrations resulted in thin filaments. By contrast, rapid gelation at higher polymer concentrations led to the formation of fibres with irregular thickness.

Supplementary references.

1. W. N. Sharratt, R. O’Connell, S. E. Rogers, C. G. Lopez and J. T. Cabral, *Macromolecules*, 2020, **53**, 1451-1463.
2. A. Gulati and C. G. Lopez, *ACS Macro Lett*, 2024, **13**, 1079-1083.
3. E. Arinaitwe and M. Pawlik, *Carbohydrate Polymers*, 2014, **99**, 423-431.
4. M. Pohl, G. A. Morris, S. E. Harding and T. Heinze, *European Polymer Journal*, 2009, **45**, 1098-1110.
5. M. Bohdanecky, *Macromolecules*, 1983, **16**, 1483-1492.
6. C. G. Lopez, S. E. Rogers, R. H. Colby, P. Graham and J. T. Cabral, *Journal of Polymer Science Part B: Polymer Physics*, 2015, **53**, 492-501.
7. C. G. Lopez and W. Richtering, *Carbohydrate Polymers*, 2021, **267**, 118117.
8. M. Edali, M. N. Esmail and G. H. Vatistas, *Journal of Applied Polymer Science*, 2001, **79**, 1787-1801.
9. R. Pamies, J. G. Hernández Cifre, M. del Carmen López Martínez and J. García de la Torre, *Colloid and Polymer Science*, 2008, **286**, 1223-1231.
